# Launching injury epidemiology

**DOI:** 10.1186/2197-1714-1-1

**Published:** 2014-03-20

**Authors:** Guohua Li, Charles J DiMaggio

**Affiliations:** Center for Injury Epidemiology and Prevention, Columbia University Medical Center, 622 West 168th St, PH5-505 New York, NY 10032 USA

**Keywords:** Accidents, Epidemiology, Injury, Emergency medical services, Public health, Trauma

## Abstract

Advances in injury epidemiology and prevention are among the landmark achievements in epidemiology and public health in the past century. Despite remarkable success and growth, the field of injury epidemiology did not have its own publication outlet until now. This commentary marks the debut of the new academic journal *Injury Epidemiology* and introduces the reader to the first batch of peer-reviewed manuscripts accepted for publication in this new journal.

Epidemiologic research on injury dates back to the 1930s (Godfrey 1937; Holcomb 1938), and over the past eight decades, injury epidemiology has developed into a well-established academic specialty. Early in 1980, advances in injury epidemiology were voted by members of the Epidemiology Section of the American Public Health Association as one of the 10 landmark achievements in American Epidemiology (Haddon 1980; Kraus 2014). Reductions in injury mortality from motor vehicle crashes and occupational mishaps are recognized as two of the ten great public health achievements of the 20th century and the first decade of the 21st century in the United States (Centers for Disease Control and Prevention 1999;2011). The past three decades have witnessed a remarkable growth in injury control research, training, and practice, both in scope and in depth. In the United States, the National Center for Injury Prevention and Control became an independent program of the Centers for Disease Control and Prevention in 1997. Since then, many state health departments have established programs for injury prevention activities and over a dozen universities have established injury control research centers. To address the tremendous health burden of injury morbidity and mortality at the global level, the World Health Organization in 2000 established the Department of Injury and Violence Prevention, which has produced a series of influential reports on violence, traffic injury, and childhood injury. Despite the remarkable growth in injury epidemiology, there has been no academic journal dedicated to publishing scholarly work in this scientific discipline—until now.

The launch of this new journal represents a milestone in the evolution of injury epidemiology and prevention. In addition to the need for a professional publication outlet, several factors coalesced to give birth to *Injury Epidemiology*. Among them were the publication of the reference text *Injury Research: Theories, Methods, and Approaches* (Li and Baker 2012), and the establishment of the Center for Injury Epidemiology and Prevention at Columbia University Medical Center, both in 2012. Through the production of the reference text, we had the opportunity to work with an international group of contributing authors (including some of the most accomplished injury epidemiologists), and one of the largest academic publishing houses in the world. The idea to create a new journal dedicated to the specialty of injury epidemiology grew naturally from the book project, with many of the contributing authors of the reference text serving on the inaugural editorial board (Table 1), and Springer serving as publisher of the new journal. The Center for Injury Epidemiology and Prevention at Columbia University Medical Center is one of the youngest injury control research centers funded by the Centers for Disease Control and Prevention. Establishment of the Columbia Center has provided us with the impetus, enthusiasm, and institutional support necessary for embarking on a sustained academic endeavor.

**Table 1:** **Names and Affiliations of the Inaugural Editorial Board of** ***Injury Epidemiology***

Name	Affiliation
Limor Aharonson-Daniel	Ben-Gurion University of the Negev
	Be’er Sheva, Israel
Susan P. Baker	Johns Hopkins University
*Honorary Editor*	Baltimore, Maryland, United States
Barbara Barlow	Columbia University
*Honorary Editor*	New York, New York, United States
Peter Barss	University of British Columbia
*Assistant Editor*	Vancouver, British Columbia, Canada
David Bishai	Johns Hopkins University
	Baltimore, Maryland, United States
Renan Castillo	Johns Hopkins University
	Baltimore, Maryland, United States
Shane V. Caswell	George Mason University
	Fairfax, Virginia, United States
Magdalena Cerdá	Columbia University
	New York, New York, United States
Hui Chen	Huazhong University of Science and Technology
	Wuhan, Hubei, China
Li-Hui Chen	Centers for Disease Control and Prevention
	Hyattsville, Maryland, United States
David Clark	Maine Medical Center
	Portland, Maine, United States
R. Dawn Comstock	University of Colorado at Denver
	Denver, Colorado, United States
Charles DiMaggio	Columbia University
*Associate Editor*	New York, New York, United States
Maureen S. Durkin	University of Wisconsin
	Madison, Wisconsin, United States
Caroline F. Finch	University of Ballarat
	Ballarat, Victoria, Australia
Samuel N. Forjuoh	Texas A&M University
	College Station, Texas, United States
Shannon Frattaroli	Johns Hopkins University
	Baltimore, Maryland, United States
Susan G. Gerberich	University of Minnesota
*Assistant Editor*	Minneapolis, Minnesota, United States
Robyn Gershon	University of California, San Francisco
	San Francisco, California, United States
Andrea C. Gielen	Johns Hopkins University
	Baltimore, Maryland, United States
Deborah Girasek	Uniformed Services University of the Health Sciences
	Bethesda, Maryland, United States
Jurek Grabowski	AAA Foundation for Traffic Safety
	Washington, District of Columbia, United States
David Hemenway	Harvard University
	Boston, Massachussetts, United States
Delia Hendrie	Curtin University
	Perth, Western Australia, Australia
Jamie Hosking	University of Auckland
	Auckland, New Zealand
Jonathan Howland	Boston University
	Boston, Massachussetts, United States
Paul D. Juarez	University of Tennessee
	Memphis, Tennessee, United States
Katherine M. Keyes	Columbia University
	New York, New York, United States
Jess Kraus	University of California, Los Angeles
*Honorary Editor*	Los Angeles, California, United States
Barbara Lang	Columbia University
*Managing Editor*	New York, New York, United States
Jean A. Langlois Orman	US Army Institute of Surgical Research
*Assistant Editor*	Fort Sam Houston, Texas, United States
Guohua Li	Columbia University
*Editor-in-Chief*	New York, New York, United States
Andrew E. Lincoln	MedStar Health Research Institute
	Hyattsville, Maryland, United States
David A. Lombardi	Liberty Mutual Research Institute for Safety
	Hopkinton, Massachussetts, United States
Becky P.Y. Loo	University of Hong Kong
	Pokfulam, Hong Kong, China
Francisco J. López-Valdés	Universidad de Zaragoza
	Zaragoza, Spain
Stephen W. Marshall	University of North Carolina at Chapel Hill
*Assistant Editor*	Chapel Hill, North Carolina, United States
Thelma Mielenz	Columbia University
	New York, New York, United States
Matthew Miller	Harvard University
*Assistant Editor*	Boston, Massachussetts, Unites States
Lenora Olson	University of Utah
	Salt Lake City, Utah, United States
Joyce Pressley	Columbia University
	New York, New York, United States
Marizen Ramirez	University of Iowa
	Iowa City, Iowa, United States
Leon Robertson	Yale University
*Honorary Editor*	New Haven, Connecticut, United States
Carol W. Runyan	University of Colorado at Denver
	Denver, Colorado, United States
Maria Seguí Gómez	University of Navarra
	Pamplona, Spain
Anbedaw W. Selassie	Medical University of South Carolina
	Charleston, South Carolina, United States
Dennis F. Shanahan	Injury Analysis, LLC
	Carlsdbad, California, United States
Gordon Smith	University of Maryland
	Baltimore, Maryland, United States
Catherine Stayton	New York City Department of Health and Mental Hygiene
	New York, New York, United States
David Sugerman	Centers for Disease Control and Prevention
	Chamblee, Georgia, United States
Jennifer Taylor	Drexel University
	Philadelphia, Pennsylvania, United States
Anna E. Waller	University of North Carolina at Chapel Hill
	Chapel Hill, North Carolina, United States
Margaret Warner	Centers for Disease Control and Prevention
	Hyattsville, Maryland, United States
Huiyun Xiang	The Ohio State University
	Columbus, Ohio, United States
Motao Zhu	West Virginia University
	Morgantown, West Virginia, United States

The editorial mission of *Injury Epidemiology* is to advance the science and practice of injury prevention and control through timely publication and dissemination of peer-reviewed research. An open-access academic journal, Injury Epidemiology aims to be the premier venue for communicating epidemiologic studies of unintentional and intentional injuries, including, but not limited to, morbidity and mortality from motor vehicle crashes, drug overdose/poisoning, falls, drowning, fires/burns, iatrogenic injury, suicide, homicide, assaults, and abuse. Relevant studies are investigations designed to understand the magnitude, distribution, determinants, causes, prevention, diagnosis, treatment, prognosis, and outcomes of injuries in specific population groups, geographic regions, and environmental settings (e.g., home, workplace, transport, recreation, sports, and urban/rural). Of special interest are studies that strengthen the scientific foundation of injury prevention and control and generate objective and practical knowledge to reduce injury morbidity and mortality on a population level. Priority consideration will be given to manuscripts that feature contemporary theories and concepts, innovative methods, and novel techniques as applied to injury surveillance, risk assessment, development of effective interventions, and program/policy evaluation.

The call for submissions was first issued in December 2013. Within three months, we received over 20 manuscripts. The first batch of manuscripts was accepted for publication following rigorous peer-review and revisions, and covers a variety of contemporary topics and innovative methods. Using innovative sampling and recruitment methods and online data collection techniques, Tefft et al. (2014) present survey results that quantify the sizable racial disparities in delayed driving licensure among 18-year-olds and that dispel the widely held misconception of graduated driver licensure programs being the main reason for delayed licensure.

Falls have long been recognized as a major cause of disability and mortality in injury epidemiology but did not receive adequate attention from government agencies and healthcare providers until recent years. The study by Stevens et al. (2014) provides valuable data for better understanding the circumstances of falls occurring in residential settings and for designing out the risk in the bathroom for older residents.

Firearm-related injury remains an important and contentious subject in the United States. Although injury researchers have produced compelling epidemiologic evidence that gun ownership is associated with a substantially increased risk of suicide and homicide, there is lingering doubt about the causality of the relationship. In a meticulously performed analysis of national data, Opoliner et al. (2014) show that the strong association between gun ownership and suicide risk is specific to suicide committed by means of firearms and is independent of depression (as indicated by antidepressant prescriptions), thus ruling out one of the putative confounders.

Injuries, both intentional and unintentional, affect American Indians/Alaska Natives (AI/AN) more than any other racial and ethnic groups. The excess risk of injury in AI/AN represents one of the most pronounced and understudied health disparities. To facilitate injury control research and practice in this underserved population, Sapra et al. (2014) provide a comprehensive review of the epidemiologic literature on interpersonal violence in AI/AN by benchmarking the prevalence of child abuse, violence against women, and elder abuse, identifying the risk factors, and outlining the research gaps.

Of special interest are the commentary pieces penned by two pioneers in injury epidemiology and honorary editors of the journal (Baker 2014; Kraus 2014). Professor Baker treats us with several personal stories that illuminate the importance and power of intuitions, first-hand observations, nuances of data, and descriptive epidemiology; and Dr. Kraus reminisces over his outstanding career and remarkable journey to injury epidemiology. We hope that our readers find these commentaries insightful and inspiring.

Establishing an academic journal is no small undertaking. If it takes a village to raise a child, it must take an international community to establish an academic journal. We are indebted to Ms. Beryl A. Abrams, Ms. Kathryn Pope, Dr. Margaret Wood, and Dr. Sandro Galea of Columbia University, and Marc Strauss, Hannah Bracken, Jon Gurstelle, and Jerson Baneal of Springer for their assistance and encouragement. Without their support, this journal would not be launched on schedule. Our heartfelt thanks also go to the editorial board members for their service and cooperation, to the reviewers for their expertise and contributions to this labor of love, and to current and prospective authors for their trust and ingenuity. The future of *Injury Epidemiology* will depend on the editorial board to safeguard its academic integrity and mission, the reviewers to ensure its scientific rigor and quality, and the authors to sustain its vitality and utility.

## References

[CR1] Baker SP (2014). The stories behind the statistics. Inj Epidemiol.

[CR2] Centers for Disease Control and Prevention (1999). Ten great public health achievements–United States, 1900–1999. MMWR.

[CR3] Centers for Disease Control and Prevention (2011). Ten great public health achievements– United States, 2001–2010. MMWR.

[CR4] Godfrey ES (1937). Role of the health department in the prevention of accidents. Am J Public Health Nations Health.

[CR5] Haddon W (1980). Advances in the epidemiology of injuries as a basis for public policy. Public Health Reports.

[CR6] Holcomb RL (1938). Alcohol in relation to traffic accidents. JAMA.

[CR7] Kraus JF (2014). A Journey to and through injury epidemiology. Inj Epidemiol.

[CR8] Li G, Baker SP (2012). Injury Research: theories, methods, and approaches.

[CR9] Opoliner A, Azrael D, Barber C, Fitzmaurice G, Miller M (2014). Explaining geographic patterns of suicide in the US: the role of firearms and antidepressants. Inj Epidemiol.

[CR10] Sapra KJ, Jubinski SM, Tanaka MF, Gershon RR (2014). Family and partner interpersonal violence among American Indians/Alaska Natives. Inj Epidemiol.

[CR11] Stevens JA, Mahoney JE, Ehrenreich H (2014). Circumstances and outcomes of falls among high risk community-dwelling older adults. Inj Epidemiol.

[CR12] Tefft BC, Williams AF, Grabowski JG (2014). Driver licensing and reasons for delaying licensure among young adults 18–20, United States, 2012. Inj Epidemiol.

